# Continuous action with a neurobiologically inspired computational approach reveals the dynamics of selection history

**DOI:** 10.1371/journal.pcbi.1011283

**Published:** 2023-07-17

**Authors:** Mukesh Makwana, Fan Zhang, Dietmar Heinke, Joo-Hyun Song

**Affiliations:** 1 Brown University, Providence, Rhode Island, United Kingdom; 2 University of Birmingham, Birmingham, United Kingdom; Johns Hopkins University, UNITED STATES

## Abstract

Everyday perception-action interaction often requires selection of a single goal from multiple possibilities. According to a recent framework of attentional control, object selection is guided not only by the well-established factors of perceptual salience and current goals but also by selection history. Yet, underlying mechanisms linking selection history and visually-guided actions are poorly understood. To examine such interplay and disentangle the impact of target and distractor history on action selection, we employed a priming-of-popout (PoP) paradigm combined with continuous tracking of reaching movements and computational modeling. Participants reached an odd-colored target among homogeneous distractors while we systematically manipulated the sequence of target and distractor colors from one trial to the next. We observed that current reach movements were significantly influenced by the interaction between attraction by the prior target feature and repulsion by the prior distractor feature. With principal component regression, we found that inhibition led by prior distractors influenced reach target selection earlier than facilitation led by the prior target. In parallel, our newly developed computational model validated that current reach target selection can be explained best by the mechanism postulating the preceded impact of previous distractors followed by a previous target. Such converging empirical and computational evidence suggests that the prior selection history triggers a dynamic interplay between target facilitation and distractor inhibition to guide goal-directed action successfully. This, in turn, highlights the necessity of an explicitly integrated approach to determine how visual attentional selection links with adaptive actions in a complex environment.

## Author summary

Most real-world visual scenes are complex and crowded, where multiple objects compete for attention and goal-directed action. The interactions between mechanisms of attentional selection and action selection are at the root of many complex behaviors. However, their link has been understudied. To examine this interplay and disentangle the impact of target and distractor history on action selection, we employed a priming-of-popout (PoP) paradigm combined with continuous tracking of reaching movements and computational modeling. This integrated approach constitutes a significant departure from existing practice, where attention and action selection mechanisms are typically investigated separately. Our converging evidence supports the notion that prior selection history triggers a dynamic interplay between target facilitation and distractor inhibition to guide goal-directed action successfully. This finding highlights the necessity of an explicitly integrated approach to determine how visual attentional selection links with adaptive actions in complex environments. Taken together, our study provides valuable insights into the mechanisms underlying attentional selection and action selection, and has important implications for future research in this field.

## Introduction

In a complex and dynamic environment, survival depends not only on our ability to select relevant information amongst distractors but also on selecting appropriate actions. Successful interactions with such complex environments require seamless coordination between mechanisms of *attentional selection* that help us make sense of the world and those that underlie *action selection*, allowing us to generate appropriate adaptive movements. How attentional mechanisms select a single object as a target has been typically framed in terms of a dichotomy between top-down control by the current goals and bottom-up control by the physical salience of objects. However, the role of past experiences, also known as selection history, has recently gained popularity as a third factor guiding attention [1–3]. In everyday activities, people need to perceive, look at, and reach target objects to interact with them and achieve their goals. If we do not consider the full scope of vision, we may overlook key processes that determine visually-guided actions. Similarly, if we view the motor system as simply executing decisions based on visual processing, we may miss important aspects of motor function [4–6]. Recent studies have underscored the value and necessity of combining visually guided actions with traditional psychophysical approaches to fully understand how we integrate perception and action to accomplish behavioral goals and resolve competing internal processes in complex visual environments [4–7].

For instance, Moher et al. [8] examined the impact of salient distractors on action selection and discovered an unexpected dissociation between action selection and visual attention. They showed that the same external information, such as perceptual salience or the associated value of a stimulus, can trigger a suppression mechanism for action but not for attention: higher contrast or high-valued stimuli were more distracting in perceptual tasks, whereas they were less disruptive when quick motor actions were required. Such distinction between attentional and goal-directed action selection cannot be captured by conventional approaches, in which these two processes are studied separately. Moher and Song [9] also supported that earlier perceptual and cognitive processing before decision "leaks" into motor systems. They showed that if the target color is repeated in consecutive trials, it results in faster target selection. Conversely, if the target color is switched, it leads to slower target selection even if the type of action is changed (e.g., switching from a saccade to a reach or vice versa). Such transfer suggests that the eyes and hands rely on a shared representation of selection history that biases attention towards or away from specific features. In parallel, recent modeling efforts have characterized such leakage between cognitive and motor processes as a simple product of neurobiologically-plausible processes where target selection and movement production processes operate in parallel [10,11]. Together, these empirical and computational findings cannot be explained by the conventional "modular" approach, assuming a functional architecture of serial information processing stages categorized as perceptual, cognitive, and motor control modules [4–6].

**Fig 1:**
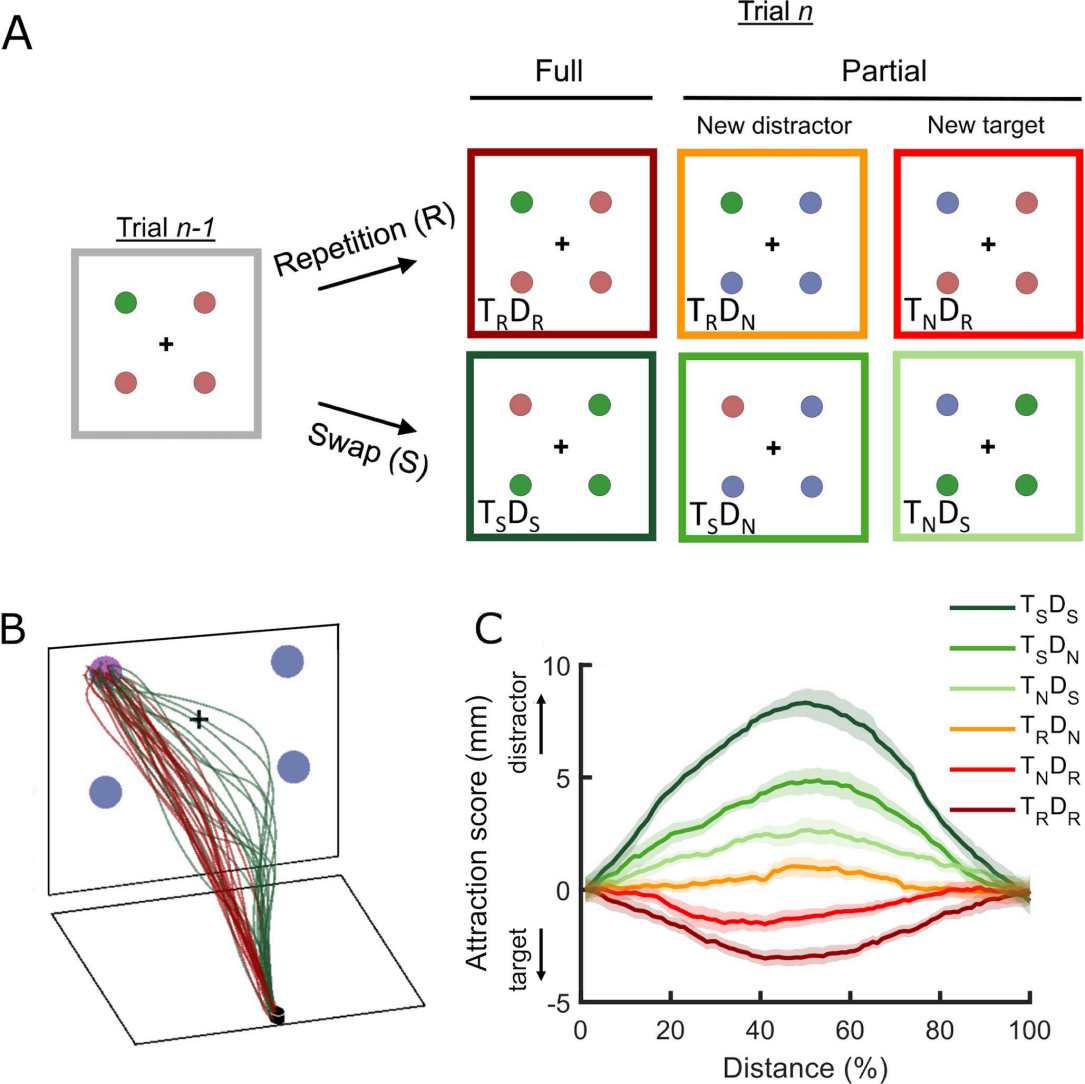
Schematic of the color-oddity task, sample reach trajectories, and behavioral results. **(A)** Schematic display sequences for different types of experimental conditions. An odd-colored target is presented with three homogeneously colored distractors in the color-oddity task. Participants search for and reach toward the odd-colored target with their index fingers. The colors of the target and distractors are pseudo-randomly selected from a pool of four colors (red, green, blue, and purple). The target location is randomly selected from one of the four corners of the imaginary square. We plotted the subset of color feature combinations inside a color-coded frame with a condition label for the demonstration purpose only. Experimental conditions are created depending on whether both (full) or one of the two-color features (partial) from a previous trial (Trial n-1) reappeared in a current trial (Trial n) and whether the reappeared color feature served the repeated (R) or swapped (S) target/distractor role. Each condition is labeled to indicate whether the color of the current target (T) or distractor (D) is repeated (_R_), swapped (_S_), or new (_N_) concerning the previous trial. For instance, in the T_R_D_N_ condition, while the color of the target is repeated from the previous one, that of distractors is new. In the T_N_D_S_ condition, while the color of the target is new, that of distractors is swapped from a previous target color. **(B)** Examples of reach movement trajectories from one participant. Curved reach trajectories from the full swap (T_S_D_S_, dark green) condition are contrasted with direct movements from the full repeat condition (T_R_D_R,_ dark red). **(C)** Attraction scores for all six experimental conditions. The positive value of the attraction score represents hand movement toward the distractors, whereas the negative value represents hand movement toward the target. The shaded region represents the within-subject standard error of the mean. Color codes are identical to Fig 1B.

Here, we aimed to unify attentional and action selection in the context of selection history by adopting an integrative approach. We combined the priming of pop-out (PoP) paradigm requiring a color-oddity selection with tracking of continuous reach movement, while implementing a neurobiologically-plausible model of attention and reaching (i.e., Selection History (SH)-Continuous Reach (CoR) model). Many studies on selection history have predominantly emphasized the role of target facilitation, but there is also considerable evidence supporting the role of distractor inhibition [12–25]. To disentangle the impact of prior history of target and distractor processing on current action selection, a color-oddity task was modified by adding partial repetition and partial swap conditions to the conventional full repeat conditions, where both target and distractor colors are repeated (T_R_D_R_, dark red border), and full swap (T_S_D_S_, dark green border) conditions, where target and distractor colors are swapped (Fig 1A). In partial repetition conditions, either target (T_R_D_N_, orange border) or only the distractor (T_N_D_R_, red border) color was repeated from a previous trial while the counterpart was in a new color. In partial swap conditions, either the target was swapped with a previous distractor (T_S_D_N_, green border) or only distractors (T_N_D_S_, light green border) were swapped with a previous target color.

**Fig 2:**
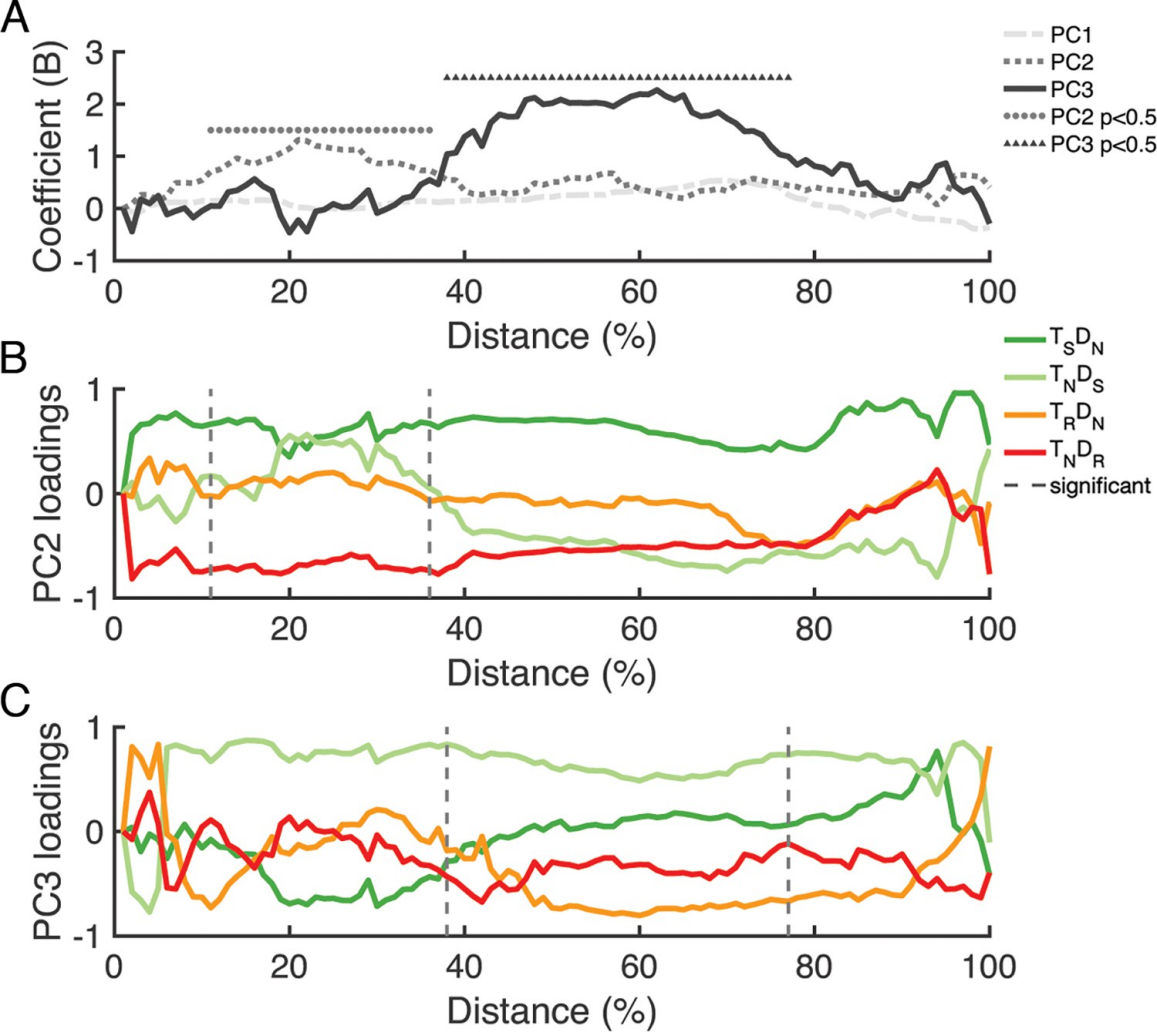
Results of the principal component regression (PCR) analysis. **(A)** The regression coefficient (*B*) of the principal components predicts PoP. The three PCs are represented by dashed, dotted, and solid plots. Circle and triangle markers represent the significant reach distances (p<.05) in the early phase (11–36% distance) for PC2 and late phase (38–77% distance) for PC3, respectively. **(B)** Factor loadings of PC2. **(C)** Factor loadings of PC3. The distances between two vertical dashed lines correspond to the distances found significant in regression. PC2 was positively loaded by T_S_D_N_ and negatively by T_N_D_R_ in the early phase (between vertical dashed lines in Fig 3B), both containing prior distractor color features. PC3 was positively loaded by T_N_D_S_ and negatively by T_R_D_N_ in the late phase (between vertical dashed lines in Fig 2C), containing prior target color features. See supplementary information for the factor loadings of PC1 and the eigenvalues (Figs A and B in S2 Text).

Using this paradigm, we first evaluated empirical evidence of prior target facilitation and distractor inhibition on current goal-directed action. We examined whether the magnitude of reaching movements deviated either toward distractors or a target in comparison with trials without features associated with the immediate history, which demonstrates the selection history effect for each condition (Fig 1C). Next, to further differentiate and quantify the contributions of previous target and distractor history to the overall PoP effect, we conducted principal component regression (PCR) analysis [26] (Fig 2). PCR analysis showed that the repetition of previous distractor features is linked to the early stages of reaching, while the repetition of previous target features is associated with the later stages. This suggests that selection history affects target selection through initial inhibition followed by subsequent facilitation.

**Fig 3:**
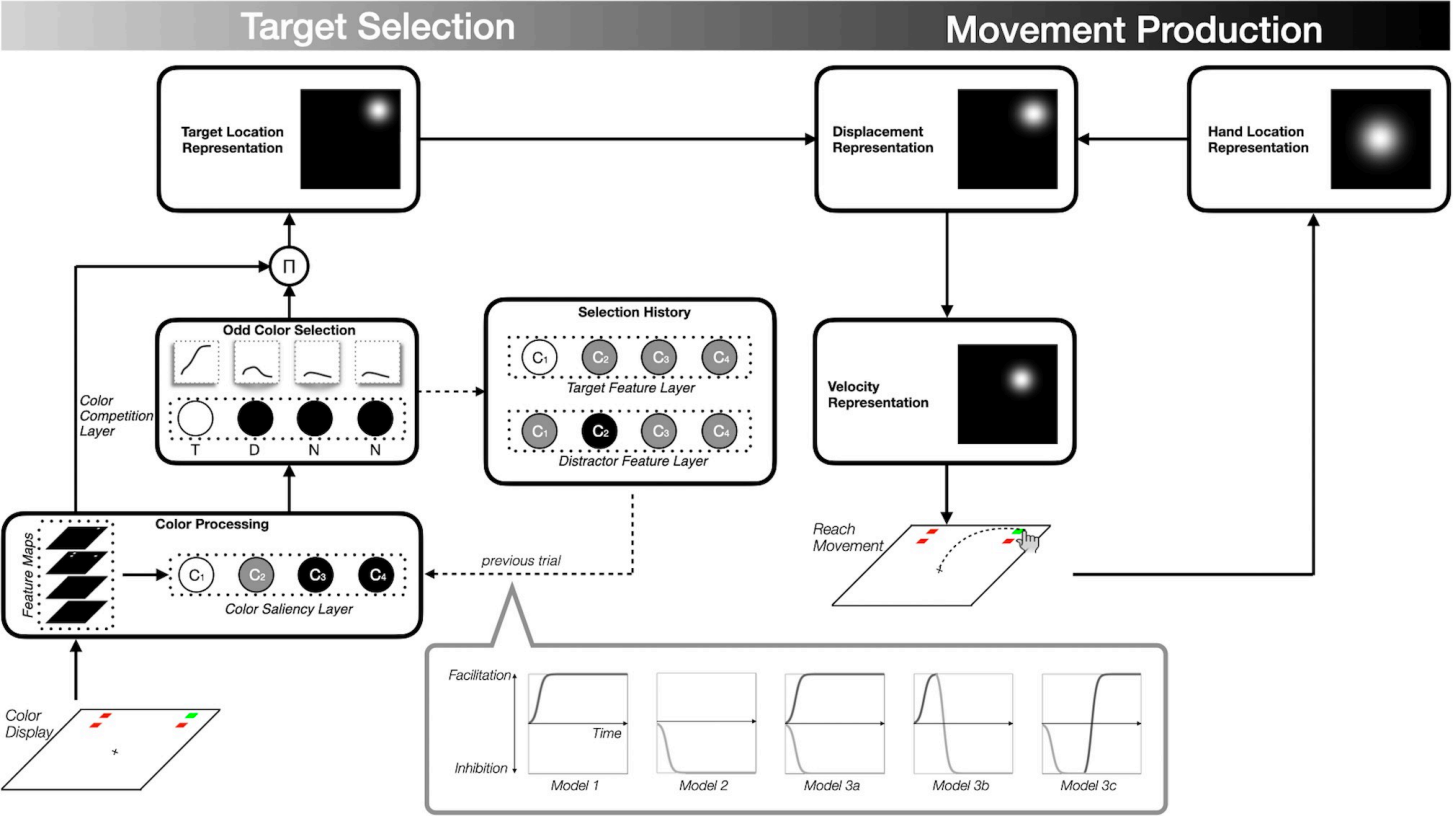
An overview of SH-CoR architecture. The model consists of two processes: Target Selection (left) and Movement Production (right). Each process comprises several modules (boxes with solid black lines). The Target Selection process consists of layers of model neurons (illustrated dotted lines, as circles C1, C2, C3, and C4 for four colors used in the experiment) in the modules, modeling the neuron-like responses to the four colors. The shading of the nodes illustrates the level of activation for the current trial, with white being the highest activation and black the lowest. Both processes and their modules operate in parallel (analogous to brain regions), allowing SH-CoR to produce the leakage effect. The first stage of Target Selection (Color Processing module) determines color Feature Maps and the saliency of the colors present in the Color Display using different color units (Color Saliency Layer). For instance, in the above display, C_1_ (white circle) and C_2_ (gray) represent the most and least salient colors in the display, while C_3_ and C_4_ (black circles) represent colors absent in the display. Based on the output of the Color Saliency Layer (see S3.A and Eq. S1 in S3 Text), the Odd Color Selection module selects the most salient color. It identifies the distractor and absent colors via color competition (Eq. S2 in S3 Text). These feature units are assigned labels, T (target color), D (distractor color), and N (absent color). The line graphs above the units remind us that color competition is a temporal process (not instantaneous) whose speed is proportional to the color saliency. These temporal activations are combined with the output of the Feature Maps in a multiplicative way (Eq. S3 in S3 Text). Initially, all possible item locations compete to become the target location, but the color competition ensures that the salient item dominates the location competition. This way, the feature map of the winning color (odd-color) eventually dominates the input to a competition of locations, generating the Target Location Representation (Eq. S4 in S3 Text). In addition, the Target Selection is also influenced by the Selection History module. This process stores the target and distractor features from the previous trial (see dotted lines; Eq. S6 in S3 Text) in separate layers. The layers in the Selection History module, in turn, influence the selection of the current target through facilitation and/or inhibition mechanism depending on which of the five models of inter-trial selection history are implemented in a particular instantiation of SH-CoR (see the gray dialog box).

In parallel, we have developed a new computational model called SH-CoR (Fig 3), building on our previous work with CoRLEGO (Choice Reaching with a LEGO arm robot). This model simulates reach trajectories and incorporates both facilitatory and inhibitory selection history mechanisms [11], as well as competitive selection mechanisms commonly used in selective attention models [27–30]. With SH-CoR, we can evaluate the effects of three different selection history mechanisms: target facilitation only, distractor inhibition only, or both. When both mechanisms are involved, we can examine whether they operate simultaneously or sequentially. Our results provide converging empirical and computational evidence that the inhibitory impact of previous distractors is followed by the facilitatory impact of a previous target during target selection for action.

## Results

### Behavioral analysis: Spatio-temporal impacts of selection history on reach movements

In this study, we primarily focused on the target-defining feature priming effect (i.e. color). In prior studies, we showed that other features such as target location-based repetition are much weaker and unstable [31]. Overall, the participants accurately (92.5% ±1.4) performed the color-oddity task, in which they were required to reach to touch an odd-colored target among homogeneous distractor, without any significant difference between the experimental conditions (see Table 1) (*F*_(6,120)_ = 0.694, *p* = .655, $\eta_{p}^{2}$ = 0.034, *η_G_* = 0.003).

**Table 1:** Display the mean (SE) for behavioral performance in the color-oddity task for the six experimental conditions with one baseline condition.

*Conditions*	*Accuracy (%)*	*Initiation latency (ms)*	*Movement time (ms)*	*Total time (ms)*
Full repeat (T_R_D_R_)	93.07 (1.38)	315.87 (10.69)	489.20 (14.05)	805.07 (20.31)
Target repeat (T_R_D_N_)	92.83 (1.48)	315.53 (11.09)	493.65 (14.24)	809.18 (20.41)
Distractor repeat (T_N_D_R_)	92.31 (1.51)	318.07 (11.20)	493.62 (14.36)	811.69 (20.73)
Target swap (T_S_D_N_)	92.27 (1.52)	315.43 (11.34)	497.41 (13.49)	813.20 (19.75)
Distractor swap (T_N_D_S_)	92.95 (1.45)	317.43 (11.04)	495.02 (14.26)	812.46 (20.45)
Full swap (T_S_D_S_)	92.23 (1.42)	316.20 (11.06)	499.14 (13.05)	815.34 (19.06)
No repeat, no swap (T_N_D_N_)	92.31 (1.49)	318.00 (11.01)	494.13 (13.76)	812.13 (19.97)

#### PoP during reach target selection

To establish the validity of our paradigm, we first focused on the performance in the two standard conditions, i.e., full swap (T_S_D_S_) and full repeat (T_R_D_R_) conditions. For instance, in prior studies where humans and non-human primates perform a color-oddity task, both saccades and reach to the target are facilitated in the T_R_D_R_ condition, i.e., reaches are completed relatively quickly and are less deviated. In contrast, in the T_S_D_S_ condition, initial saccades and reaches are directed toward a distractor more often and subsequently corrected in-flight to the target (i.e., more curved trajectories) [9,30,31].

In accord, we first observed that participants moved (movement time, *t*(20) = 4.16, *p* < .001, *d* = 0.9) and completed the reach (total time, *t*(20) = 3.42, *p* = .003, *d* = 0.747) faster in T_R_D_R_ compared to the T_S_D_S_ condition, though they initiated movements similarly (initiation latency, *t*(20) = 0.217, *p* = .83, *d* = 0.047) (see Table 1). We also observed that movement trajectories deviated more toward distractors in the T_S_D_S_ (dark green trajectories) than in the T_R_D_R_ condition (dark red trajectories) (Fig 1B). We calculated average attraction scores across participants to understand how action selection evolves as the hand moves toward the target. Attraction scores indicate how far reach trajectories deviate toward a target (represented as a negative value) or away from the target (i.e., toward distractors; represented as a positive value) in comparison with trials without features associated with immediate history (T_N_D_N_) (see Methods: Procedure). Fig 1C depicts attraction scores as a function of normalized reach distance. A cluster-based permutation test [32] (see Methods for details) revealed that trajectories were attracted more toward the distractors in the T_S_D_S_ (dark green) from 2% to 93% of reach distance and were attracted toward the target in the T_R_D_R_ condition (dark red) from 10% to 79% of reach distance. Consequently, we observed a significant difference between the attraction scores from these two conditions (2–94%), characterizing the PoP effect reflected in reach trajectories.

To summarize, the PoP effect in spatio-temporal domains shows that perceptual history can significantly enhance the efficiency of target selection for action, which reduces the occurrences of a redirected movement from a distractor to the target in accordance with previous studies [33,34]. We also compared the priming effect for full vs. partial conditions in a separate analysis. As expected, the overall priming effect was stronger for full conditions than for partial conditions (Fig A in S1 Text).

#### Effects of recent experience of the target and distractor features on reach target selection

We examined the separate contribution of target facilitation and distractor inhibition for reach target selection based on the partial repetition and partial swap conditions. First, we delved into whether repetition of a previous target (T_R_D_N_) or previous distractor feature (T_N_D_R_) differently influences reach target selection. Overall, participants showed similar movement time (T_R_D_N_ vs. T_N_D_R_, *t*(20) = 0.017, *p* = .987, *d* = 0.004) and total time (T_R_D_N_ vs. T_N_D_R_, *t*(20) = 1.381, *p* = .183, *d* = 0.301) in both conditions. However, participants initiated the reach relatively faster in the T_R_D_N_ compared to the T_N_D_R_ condition (*t*(20) = 2.29, *p* = .032, *d* = 0.502) (see Table 1). The cluster-based analysis of attraction score revealed that these two conditions significantly diverge from 23% to 70% of reach distance, where T_N_D_R_ has a larger attraction. Thus, it appears that previous distractor feature repetition has a stronger impact on reach target selection.

Next, we examined whether the previous distractor becoming the current target (T_S_D_N_) or the previous target becoming the current distractor (T_N_D_S_) differently affects reach target selection. Overall both conditions did not differ significantly in terms of initiation latency (*t*(20) = 1.192, *p* = .247, *d* = 0.26), movement time (*t*(20) = 1.65, *p* = .114, *d* = 0.361) or total time (*t*(20) = 0.472, *p* = .642, *d* = 0.103) (see Table 1). However, attraction scores (Fig 1B) indicate that reach trajectories were significantly swayed toward the distractors regardless of whether the target (T_S_D_N_, green, 4–82% distance) or distractor (T_N_D_S_, light green, 12–84%) were swapped, which is confirmed by the cluster-based permutation test. Interestingly, the deviation in the T_S_D_N_ condition was more pronounced than in the T_N_D_S_ condition (21–67%), suggesting that the previous distractor that became the current target had a stronger influence than the previous target that became a distractor. This is also consistent with the prominent role of previous distractors in the partial repetition (T_N_D_R_) condition, as seen above.

It is worth noting that the reach attraction toward the target in the T_N_D_R_ condition and toward the distractor in the T_S_D_N_ condition can be attributed to the distractor inhibition from previous distractors. Similarly, the reach attraction toward the target in the T_R_D_N_ condition and toward the distractor in the T_N_D_S_ condition can be attributed to the target activation from the previous target. While we observed that the reappearance of the previous target features influences target selection, we consistently observed that the previous distractor feature has a stronger impact, regardless of whether it serves the same distractor role (T_N_D_R_) or swapped to the target role (T_S_D_N_) in the current trial. These results suggested that inhibition from previous distractor features and facilitation from previous target features are likely to contribute to PoP during target selection. However, it is challenging to dissociate and systematically evaluate the relative contribution of the previous target and distractor to PoP over time solely based on conventional behavioral analysis. In the next section, thus, we applied the principal component regression analysis (PCR) to attraction scores across the distance. This approach can reveal the temporal dynamics of the PoP effect led by previous target facilitation and distractor inhibition while avoiding any collinearity arising from the nature of evolving movement kinematics across conditions.

### Principal component regression (PCR) analysis: Dissociating relative contributions of previous target and distractor features in selection history

With PCR analysis, we intended to determine whether the linear composite of the partial conditions (T_R_D_N_, T_N_D_R_, T_S_D_N_, T_N_D_S_) extract *interpretable* new variables (principal components, PCs) representing previous or current target and distractor features and whether we can explain the PoP effect using these PCs. First, PCR analysis extracted the most important PCs in the attraction scores of the four partial conditions (Fig 1C) using principal component analysis (PCA). Then the obtained PCs were regressed to the PoP effect, i.e., attraction score difference between T_S_D_S_ and T_R_D_R_ conditions across the reach distance. This allows us to interpret further each PC’s relative contributions to spatio-temporal dynamics in PoP. Fig 3A depicts the regression coefficients (*B*) of the first three PC: PC1 (dashed), PC2 (dotted), and PC3 (solid line) across the reach distance. The first three PCs, which explained 95% of the variance at each distance, were used for further regression analysis (Fig B in S2 Text). Note that we reported an averaged regression coefficient B (± standard deviation) for each PC and the mean-variance R^2^ (± standard deviation) of the regression model. As shown in Fig 3A, the regression analysis revealed that PC2 (dotted line) significantly predicted PoP (*R*^*2*^ *= 0*.*35±0*.*06*, *B = 0*.*97±0*.*18*) in the early phase (11–36%, marked by a circle) while PC3 (solid line) significantly predicted PoP (*R*^*2*^ *= 0*.*52±0*.*11*, *B = 1*.*77±0*.*37*) in the late phase of reach movements (38–77%, marked by triangle). PC1 (dashed line) did not significantly predict the priming effect at all distances (*p = 0*.*39*, *B = 0*.*19±0*.*17*).

Then, we examined PC loadings of four partial conditions during the significant reach phase identified by the regression analysis: the early phase of PC2 (11–36%) in Fig 3B and the late phase of PC3 (38–77%) in the Fig 2C. We focused on partial conditions that loaded beyond the cut off value of 0.35, which is conventionally considered meaningful and provides a consistent and reasonable criteria for evaluating all conditions [35–37]. To ensure the validity of our results, we also applied rigorous statistical criteria to avoid overinterpretation: a factor loading must exceed the cut-off for at least 15% of consecutive distances to be considered significant.

In the early phase of PC2 (Fig 2B), T_S_D_N_ (green) and T_N_D_R_ (red) were considered: T_S_D_N_ (green) was loaded positively (*M = 0*.*61*, *ranging from 0*.*35 to 0*.*76*), whereas T_N_D_R_ (red) was loaded negatively (*M = -0*.*70*, *ranging -0*.*77 to -0*.*58*). In these two partial conditions, prior distractor color reappeared in the current trials while serving the same in the T_N_D_R_ but reversed in the T_S_D_N_. In both, attraction scores (Fig 1C) show that reaches were more swerved away from the stimulus sharing a previous distractor feature (i.e., distractors in T_N_D_R_ and a target in T_S_D_N_). Overall, it is consistent with the idea that suppressed previous distractor features can result in the repulsion of reach movements. Therefore, we postulated that PC2 reflects inhibition from the previous distractor features, which primarily manifests in the early phase.

In the late phase of PC3 (Fig 2C), the T_N_D_S_ (light green) and T_R_D_N_ (orange) conditions were loaded positively (*M = 0*.*63*, *ranging 0*.*49 to 0*.*83*) and negatively (*M = -0*.*62*, *ranging -0*.*80 to -0*.*36*, after 42% distance), respectively. In contrast to the early phase of PC2 loaded by T_S_D_N_ (green) and T_N_D_R_ (red) conditions, including the reappearance of the prior distractor features, the late phase of PC3 is loaded primarily by the other two partial conditions, T_N_D_S_ and T_R_D_N_. In these two partial conditions, the prior target color reappeared in the current trial while serving the same in the T_R_D_N_ but reversed in the T_N_D_S_ condition. In both, attraction scores (Fig 1C) demonstrated that reaches were relatively attracted toward the stimulus sharing a previous target feature (i.e., a target in T_R_D_N_ and distractors in T_S_D_N_). It is in accord with the notion that facilitated previous target features can attract reach movements. Therefore, we postulated that PC3 reflects facilitation from the previous target features. Hence, it is plausible to primarily align PC2 with an early phase inhibitory influence from the previous trial and PC3 with a late phase facilitatory influence from the previous trial.

To summarize, PCR results further appear to narrow down the exact mechanisms of selection history influencing action, suggesting that impacts from previous target features are preceded by previous distractor features. However, because PCR analysis only assumes linear relationships between conditions, it may not fully capture non-linear effects of selection history modulating reach movements. To obtain stronger converging evidence, we implemented SH-CoR, a computational model based on well-established biological mechanisms involved in attentional selection and movement production. This allowed us to directly evaluate whether the mechanism of selection history suggested by PCR (i.e., target facilitation preceded by distractor inhibition) or an alternative mechanism is most likely to drive our behavioral findings under plausible biological constraints.

### Computational modeling with SH-CoR: Extracting mechanisms underlying selection history via neurobiologically-plausible mechanistic constraints

To link biological constraints with continuous reach data from humans, here, we simulated human performance with SH-CoR and determined which model could explain the impact of the history selection for reach target selection the best.

SH-CoR’s architecture consists of two processes (Fig 3): Target Selection (left) and Movement Production (right). The Movement Production process generates reaching movements toward the odd-colored target as localized by the Target Selection process. The target is localized based on the Odd Color Selection module, which is influenced by the Selection History module. The Selection History module stores the target and distractor features from the previous trial in separate layers (dotted lines). The layers in the Selection History module influence the selection of the current target through facilitation and/or inhibition mechanisms.

The specific mechanism used depends on which of the five models of inter-trial selection history are implemented in a particular instance of SH-CoR. These models include target facilitation only (model 1), distractor inhibition only (model 2), and both (model 3). In model 3, we also considered three variants of temporal weightings that combine prior target and distractor features. Three variants included *simultaneous co-contribution* (model 3a), *facilitation precedes inhibition* (model 3b), and *inhibition precedes facilitation* (model 3c). Note that previous studies, which primarily employed drift-diffusion models, cannot distinguish between these alternative mechanisms because they typically do not consider separate target facilitation and distractor inhibition and primarily focus on perceptual selection history [38–40].

According to our behavioral and PCR analysis, models 1 and 2 are unlikely to be consistent with observed reaching behaviors. However, we included them as a cross-check while focusing on the three equally likely variants of model 3. For instance, if the right level of facilitation and inhibition is chosen, model 3a can successfully reproduce our findings. However, SH-CoR’s neurobiologically-plausible mechanisms impose constraints on these values, which may limit the success of the models.

Among the constraints, here, we highlighted those imposed on the Odd Color Selection module as it is the most critical module. This module is governed by the suppression of distractors through global inhibition (see coefficient d in Eq. S2 in S3 Text) and the selection of the target through self-excitation (see coefficient b in Eq. S2 in S3 Text). This asymmetry in terms of suppression and excitation suggests that whether previous features serve the same (repeat condition) or reserved role (swap condition) can result in different magnitudes of movement attraction (Fig 1C). If the biases from the Selection History module are excessive, the Odd Color Selection module creates reaches in the swap conditions that are pulled too much towards a distractor and pushed too far away from a target. To prevent such erroneous target selection, the Odd Color Selection module also constraints the magnitude of inhibition and facilitation.

**Fig 4:**
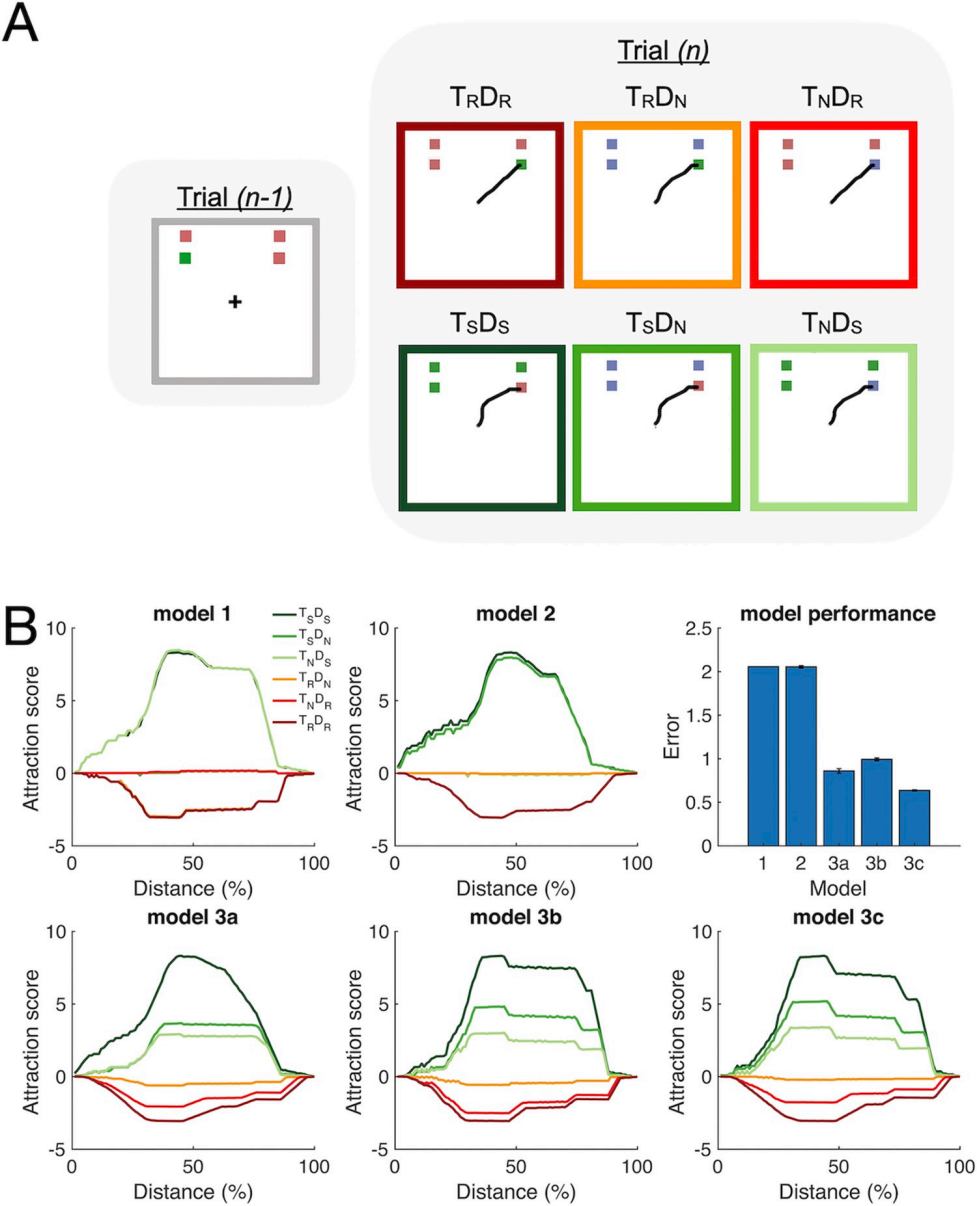
Simulation results and performance of SH-CoR modeling. **(A)** Examples of the modeling displays and simulated reaching trajectories (black lines) in a single trial. Colored squares represent targets and distractors in each display. The display in the previous trial would have the same target and distractor colors as in T_R_D_R_. Here we only show the displays and the examples of reaching trajectories for each condition in the current trial. Note that the target is always positioned at the bottom-right corner of the display for a clear illustration. **(B)** The averaged attraction scores of twenty-one repetitions for each best-fitted model with the application of noise in the color selection process and the goodness-of-fit for the five models. The parameter settings of each model can be found in Table C in S3 Text. The model performance is presented as error measurements (Eq. S16-19 in S3 Text), with error bars indicating a standard error (**Methods** and S3.D-E in S3 Text for more details).

Fig 4A illustrates the set-up of SH-CoR with the input displays of the six experimental conditions (see Method: SH-CoR). The quality of the fits was evaluated by minimizing the error between the model and mean human trajectories at each distance (see Eq. S19 in S3 Text). We found the best fitting parameter settings for each of the five alternative models (see Table B in S3 Text). Fig 4B shows the averaged attraction scores from the best fit of each model together with fitting errors. As expected from the PCR analysis and the behavioral analysis, the mean fitting error indicates that the simultaneous facilitation and inhibition model (model 3a, *error* = 0.86 ± 0.05*)* result in clearly better fits than the models which assumes facilitation only (model 1, *error = 2*.*06 ± 0*.*0001*, *t(20) = 22*.*90*, *p<0*.*001*) and inhibition-only model (model 2, *error = 2*.*05 ± 0*.*03*, *t(20) = 18*.*23*, *p<0*.*001*). Among variants of model 3, we found that model 3c (*error = 0*.*64 ± 0*.*01*) fits the best compared to model 3a (t(20) = 4.11, p<0.001) and model 3b (error = *0*.*99 ± 0*.*03*, *t(20) = 10*.*34*, *p<0*.*001*).

Our simulation clearly supported model 3c (inhibition precedes facilitation) as the best model. A close inspection of the results indicated that the two partial swap conditions, T_N_D_S_ (light green) and T_S_D_N_ (green), were critical for determining the success or failure of each model. For instance, under the constraints set by biological mechanisms (e.g., erroneous target selection), the Odd Color Selection module in model 3a (simultaneous co-contribution) was unable to generate the proper combination of simultaneously operating facilitation and inhibition processes to mimic human behavior. In contrast, Model 3b, which were not constrained by the simultaneous implementation of inhibition and facilitation processes like Model 3c, led to better simulation results than model 3a.

However, implementing facilitation first and then inhibition in model 3b resulted in outcomes that were not exhibited in human performance. For example, T_S_D_N_ had a smaller attraction score than T_N_D_S_ at the early phase of movement (Fig 4B). This was due to a mechanistic flaw in model 3b. Specifically, in T_N_D_S_, initial facilitation causes reach movements to be attracted towards a distractor in the early phase of movements. In contrast, in T_S_D_N_, due to the lack of inhibition, this attraction is minimal. In the late phase of movements, their relationship is reversed to simulate human data as closely as possible. Fig 4B represents outputs with the best-fit parameters that minimize the mismatch with human data, so the difference between T_S_D_N_ and T_N_D_S_ at the early phase appears subtle. Fig C in S2 Text illustrates an increased reversal effect with the level of facilitation and inhibition that does not provide the best-fit.

Further analysis also confirmed the robustness of model 3c, even when switching inhibition and facilitation at different times. For instance, instead of switching at 65% between the onsets of display and movement, which produced the best fit, switching at 80% still resulted in better performance (error = 0.71) than model 3b. Therefore, Model 3c and the results of PCR analysis (Fig 2) provide converging evidence supporting the preceded impact of previous distractors followed by a previous target during reach target selection.

## Discussion

The present study used an ecologically valid continuous reach tracking approach combined with neurobiologically inspired computational modeling. The results demonstrated the precedence of the distractor inhibitory process followed by target facilitation in the color-oddity task for goal-directed reaching movements. We also demonstrated that target and distractor history from the previous trial influences attentional selection, which leaks into the motor system and influences the action selection process, as reflected in the curved reach trajectories both in behavior as well as in model simulations. Selection history also encompasses past experiences associated with rewards, aversive stimuli, perceptual features, or statistical learning [12,41–43]. We focused on how the interplay between facilitation and inhibition of previous perceptual features guides intertrial priming during target selection for goal-directed action.

First, the present study contributes to long-standing debates in understanding selection history mechanisms, especially supporting an integrated framework where perception and action dynamically interact to achieve behavioral goals. While most researchers agree upon the role of target facilitation and/or distractor inhibition governing selection history [12, 15, 22–25], there is debate regarding the level of contribution and dynamics of the target facilitation and distractor inhibition [18–20,24]. For instance, Maljkovic and Nakayama [12] suggested that target facilitation and distractor inhibition contribute to selection history while target facilitation has a stronger role. Findings based on drift-diffusion modeling further suggested that the target feature plays a critical role in the selection history [39]. Whether similar mechanisms operate when a task involves goal-directed action has not been examined.

Using SH-CoR, we compared three possible mechanistic models to explain selection history and validated that distractor inhibition contributes to the early phase of reach target selection. In contrast, target facilitation contributes more during the later phase of reach target selection. Our result of distractor inhibition preceding target activation in the reaching behavior is in accord with previous studies emphasizing the role of distractor processing along with targets [24,44]. For instance, a recent study investigating statistical learning-based selection history demonstrated the stronger role of distractor suppression [20]. Another study using event-related potentials and behavioral data showed that inhibition of distractor features, rather than activation of target features, is the primary driver of early feature-based selection, thus suggesting that inhibition plays a larger role at an earlier stage of target selection than previously recognized [19]. Moher et al. [8] also showed that action selection could trigger early suppression of both physical and reward-driven salient distractors in contrast to perceptual selection. It is worth further investigating whether the observed pattern of inhibition leading to facilitation during attentional selection in visually-guided reaching is a true computational motif or simply a reflection of the specific task’s default cost function. For example, altering the costs attributed to distractors and targets could potentially modify this particular sequence.

Second, our biologically inspired modeling approach elucidated the link between attentional and action selection (SH-CoR, Fig 2). Most computational studies on understanding selection history effects in an oddity target search experiment [38–40] have been based on Ratcliff’s diffusion model (RDM) [45]. RDM conceptualizes decision-making in such visual experiments as a noisy accumulation process of decision-relevant perceptual evidence (e.g., the color), parameterized by the rate of evidence accumulation, the response threshold determining the amount of evidence needed to generate a response, and the bias toward a certain response. Nevertheless, these models do not consider neurobiological processes such as dynamics of inhibition and facilitation. Furthermore, these studies are based on the traditional PoP, including only full repeat and full swap and requiring discrete responses (e.g., keypresses). Consequently, these studies are unlikely to address questions as to separate impacts of facilitation and inhibition in selection history and their time course of guiding goal-directed action.

By implementing a leakage mechanism simulating the interaction between target selection and movement production, SH-CoR is uniquely positioned to investigate moment-by-moment spatio-temporal dynamics driven by selection history during goal-directed action selection while confirming the basic findings by RDM. This is possible because SH-CoR’s competitive selection mechanism (e.g., Eq. S2 in S3 Text) is similar to RDM’s noisy evidence accumulation. The time constant of our model neuron can be seen as analogous to the accumulation rate, and the response threshold is implemented through parameters in the motor system. RDM’s response bias has a similar effect to our model neurons’ input (e.g., Eq. S2 in S3 Text). Critically, the RDM studies find that color priming can be best described through changes in the response bias and not by the other two parameters (see Ásgeirsson et al. [46] for an alternative account), which is consistent with SH-CoR’s realization because SH-CoR’s selection history module forms an input into Color Competition Layer. Beyond such basic PoP effect, SH-CoR can uncover mechanisms for the change of response bias, i.e., inhibition led by prior distractor feature and facilitation led by prior target feature on reach target selection (Model 3c).

One RDM-based study explored the inhibitory influence of distractors on response bias in a classic PoP experiment [38]. Allenmark et al. [38] found that a model analogous to our model 1 (facilitation-only) is superior to a combination of facilitation and inhibition (Model 3a). On the face of it, these findings seem to be inconsistent with ours. However, they did not consider the dynamic influence of facilitation and inhibition (models 3b and 3c). Our results suggest that these models could have provided a much better fit than their facilitation-only model. The difference could also stem from their experimental design lacking the partial conditions, which can provide more precisely estimated contributions of facilitation and inhibition, or their keypress-only design and our continuous reach target selection. Furthermore, one benefit of our reach paradigm is that it can reveal the time course of the facilitation and inhibition effects given that it is uses a continous measure.

Here, the SH-CoR model is optimized to explain intertrial priming showing transient short-term inter-trial effects. However, some types of selection history, such as rewards, can have a long-term effect [12, 47]. For instance, Anderson and Yantis [47] showed that reward history associated with attention capture could last up to six months. The Selection History module in the SH-CoR model can be easily extended to incorporate such long-term history effects. As such, SH-CoR can serve as an all-in-one generalized model, which provides a comprehensive framework for various selection history mechanisms guiding attentional and action selection. Furthermore, it would be also worthwhile for future studies to examine the generalizability of our finding that the history of inhibition leads to facilitation during attentional selection in visually-guided reaching. For instance, a prior study [8] demonstrated a strong resemblance between the suppression of physically salient distractors, as in our study, and reward-driven distractors during action selection.

To summarize, our findings have implications for understanding the dynamic interaction between the integrated attention-action systems in humans. Furthermore, since the SH-CoR model can easily integrate robotics which could behave on par with human-like performance, it can also lead to the development of better and more efficient human-computer interaction systems. Therefore, we urge that combining perception and action provides exceptional research opportunities that will enhance our understanding of a wide range of brain mechanisms, enabling seamless coordination of behavior in the complex world.

## Methods

### Ethics statement

The experimental protocol was approved by the Brown University Institutional Review Board in accordance with the Code of Ethics of the World Medical Association (Declaration of Helsinki) for experiments involving humans. Informed written consent was obtained from all the participants.

Behavioral experiment and data analysis methods for the current study were adapted largely from that of Moher & Song [31,48].

### Participants

Twenty-one Brown University undergraduate volunteers (five females, mean age 20.19±1.36 years) participated in this experiment for course credit. All participants were right-handed and had a normal or corrected-to-normal vision.

### Apparatus

All the stimuli were projected on a plexiglass display perpendicular to the table. The projector was placed behind the plexiglass. Observers were seated on a non-metallic chair and facing the plexiglass at approximately 57 cm from their line of vision. The three-dimensional hand position was recorded at approximately 240 Hz using an electromagnetic position and orientation recording system [Polhemus Liberty; Polhemus Inc., Colchester, VT] with a measuring error of .03 cm root mean square. The observer’s index finger rested on a Styrofoam block placed in front of them on the table, located 27 cm from the screen along the z-dimension (i.e., the axis bounded by the observer and the display). A motion tracking marker was secured with a Velcro strap near the tip of the right index finger. The finger was aligned with the bottom of the display along the y dimension (i.e., the axis bounded by the top and bottom of the display) and the horizontal midline of the display along the x-dimension. Stimulus presentation was conducted using custom software designed with MATLAB (version 2015b) [49] and Psychtoolbox [50].

### Stimuli

All stimuli were presented on a black background. Each trial began with a fixation cross at the center of the screen with a width and length of 0.7 cm (0.7° of visual angle).

### Procedure

At the beginning of the experiment, a nine-point calibration was conducted for hand position. All stimuli were presented on a black background. Participants were instructed to place their right index finger on the starting position. Each trial began with a fixation cross presented for 500ms, ensuring that the participant’s index finger remained within starting position. After fixation, four circles, each with a 2 cm diameter (2°), were presented at four corners of an imaginary square with a side of 13 cm (12.7°) (measured from center to center). The circles were rendered in either red (RGB: 195, 107, 107), green (RGB: 61, 152, 63), blue (RGB: 114, 125, 180) or purple (RGB: 177, 104, 190). All colors were approximately equiluminant using photometer calibration (red: 17.45 cd/m^2^, green: 17.36 cd/m^2^, blue: 17.45 cd/m^2^, purple 17.42 cd/m^2^). Out of four, one circle was always rendered in odd color (target) compared to the other three homogeneously colored circles (distractors). The color and location of the target were randomized across trials. Participants were instructed to reach and touch an odd-colored circle with their right index finger as quickly and accurately as possible within 1500 ms. Following every trial, participants were given auditory feedback to indicate whether their response was accurate (600Hz beep) or inaccurate (300Hz beep). The reach data were recorded for an extra 200ms once they touched the screen. The experiment began with 12 practice trials, followed by seven blocks of 120 trials each. In each block, we had approximately equal numbers of randomly mixed seven trial types based on whether the target and distractor features were repeated or swapped compared to the previous trial: full repeat (T_R_D_R_), full swap (T_S_D_S_), partial target repeat (T_R_D_N_), partial distractor repeat (T_N_D_R_), partial target swap (T_S_D_N_), partial distractor swap (T_N_D_S_), and no repeat no swap (T_N_D_N_) (Fig 1A).

### Data analysis

#### Reach data analysis

Hand movement data were analyzed using custom MATLAB (version R2021b) [49] software. Three-dimensional resultant speed scalars were created for each trial using a differentiation procedure. These scalars were then submitted to a second order, low-pass Butterworth filter with a cutoff of 10 Hz. Movement onset was calculated as the first time point on each trial after stimulus onset at which hand movement speed exceeded 15 cm/s. Movement offset was the first subsequent measurement on each trial when speed decreased to below 15 cm/s. Each individual trial was visually inspected [34]; for trials in which the default threshold clearly missed part of the movement or included substantial movement back to the starting point, thresholds were adjusted manually to more appropriate levels for that trial (<1% of all trials). All trajectories were normalized to 100 data points across reach distance using functional normalization [51,52]. Attraction scores were calculated in the following way. First, horizontal (x-dimension) and vertical (y-dimension) deviation scores were calculated for each trial by subtracting the corresponding mean trajectory for T_N_D_N_ separately for each of the four target locations.

We assigned positive signs to deviation scores if the deviation was toward the distractors and negative signs if it was toward the target. For example, for a top-left target, a rightward deviation would have a positive horizontal deviation score and a leftward deviation would have a negative score with respect to the T_N_D_N_ condition. Similarly, an upward deviation would have a negative vertical deviation score and a downward deviation would have a positive score. Therefore, this sign assignment takes into account each target location in relation to its distractors. We then combined the horizontal and vertical deviation scores using their Euclidean distance and assigned the sign of the winner of the horizontal vs. vertical to get the attraction scores. The average attraction scores across participants for each condition are shown in Fig 1C.

For all analyses, we excluded all trials with incorrect current or previous target selection, excessive reach sampling drop, or no movement. We conducted paired t-tests whenever means of two conditions were compared and used one-way repeated measures ANOVA when means of seven conditions were compared. Assumptions of normality (Shapiro-Wilk) and sphericity (Mauchly’s test) were checked, and appropriate corrections (Greenhouse-Geisser) were applied in case of violation. After conducting planned comparisons following a one-way ANOVA, we applied the Bonferroni correction for multiple comparisons. However, we only corrected for the number of planned comparisons and did not include other possible comparisons that were not performed. The significance level applies to the family of comparisons rather than each individual comparison [53,54]. Statistical analysis was conducted using JASP [55].

We used a cluster-based analysis to determine when during the reach movement the attraction scores significantly swayed toward the target or distractors [32]. Following Moher et al. [8], we calculated the t-statistic for the attraction score at each time point and searched for the largest consecutive cluster of time points where the t-statistic was above the threshold. We then calculated the sum of t-values within that cluster (the threshold was set based on the t-value for the degrees of freedom of the experiment at α = .05). Next, we randomly permuted the order of t-statistic values 100,000 times and performed the same cluster analysis on each permutation to get a distribution of possible cluster sizes. We then calculated a p-value for the observed cluster size against this distribution. If the observed cluster size was significant with p < .05, we reported the start and end points of the cluster as the points of movement affected by distractor presence.

#### Principal component regression (PCR) analysis

The PCR analysis consists of two: The data is analyzed with a principal component analysis (PCA) then the resulting principal components are regressed on a predictor variable. Applied to our data first we performed a PCA on the four partial conditions and then regressed the principal component scores on the attractor score differences between the full repeat condition and the full swap condition using a no-intercept linear regression model. We also included an additional analysis step to ensure an accurate representation of attraction scores across all distances since the sign of PCA’s principal components (PCs) is mathematically indeterminate. In other words, positive or negative PCs can be an equally valid outcome of a PCA analysis. Consequently, the sign of a PC can change as a result of small changes in the data and the numerical implementation of the PCA analysis. In a standard application of PCA, these signs do not play a role as the interpretation of the component is typically independent of the sign. However, since we applied PCA to attraction scores across all distances, changes of the sign from distance to distance misrepresent the smoothly changing attraction scores. Therefore, we compared PC loadings between two consecutive distances across 11–90%. If the dot product between the two components was larger, i.e., the angular differences between the two vectors are more than 120°, we flipped the signs of the PCs of the second distance. Note that we performed PCR between 11–90% reach distances to apply our data. The first and the last 10% distances were cut off as the attraction scores of all conditions are near zero at the beginning and the end of the reach movement (Fig 1C).

#### Computational modeling: SH-CoR

SH-CoR model implements Dynamic Neural Fields (DNFs) Theory for the Movement Production process and the Target Location Representation [56,57]. The color competition is performed using a modified Grossberg recurrent neural network [58]. SH-CoR is implemented in MATLAB (version R2019b) [49] using the COSIVINA Toolbox [57]. The equations and the parameters can be found in S3 Text.

SH-CoR used 2D movements while the display was processed from a bird’s eye view without considering moving toward the display (i.e., not considering the z-axis). It reduced the complexity of the behavioral experiment in a 3D environment (e.g., coordinate transformations, solving inverse kinematics, 3D perception, etc.) to a feasible level in terms of computational demand and complexity while maintaining critical elements to address research questions, including simulating reach trajectories. To match the analysis of human data, we also calculated attraction scores by taking the trajectories of the No-repeat-no-swap (T_N_D_N_) condition as a baseline and subtracting it from that of each condition. To find the best parameter values, we ran a grid search [59] for each model guided by a goodness-of-fit function (S3.D-E in S3 Text for details).

## Supporting information

S1 Text Priming effect comparison. Fig A. Results comparing full priming and partial priming effect.
(PDF)

S2 Text Principal component regression (PCR) analysis. Fig A. Factor loadings of PC1. Fig B. The cumulative variance explained by the three PCs across reach distance. Fig C. An illustration of a typical reversal effect between T_S_D_N_ (medium green) and T_N_D_S_ (light green) in model 3b (facilitation precedes inhibition) with non-optimal parameters.
(PDF)

S3 Text Computational Modelling. Table A. Parameter setting for color saliencies. Table B. Best fitted model parameters for the Target Selection process. Table C. Parameter ranges of the final grid search in the Target Selection process for the five models. Table D. DNFs parameters in the Movement Production process. S3.A. Target Selection process and mathematical description. S3.B. Selection History module. S3.C. Movement Production process and mathematical description. S3.D. Searching for best parameters. S3.E. Goodness-of-fit. Eq.S1 –S19.
(PDF)
