# Supplementary material for: Continuous action with a neurobiologically inspired computational approach reveals the dynamics of selection history
Source: PLoS Comput Biol. 2023 Jul 17;19(7):e1011283. doi: 10.1371/journal.pcbi.1011283 (PMC10374010; doi:10.1371/journal.pcbi.1011283)
Supplement: S1 Text — Fig A. Results comparing full priming and partial priming effect. (PDF) [file pcbi.1011283.s001.pdf]

Supporting Information S1 Text for:

Continuous action with a neurobiologically inspired computational approach reveals the dynamics of selection history

Mukesh Makwana[1,¶], Fan Zhang[2,¶], Dietmar Heinke[2], and Joo-Hyun Song[1]*

[1] Brown University, Providence, Rhode Island, USA

[2] University of Birmingham, Birmingham, United Kingdom

¶ contributed equally and are co-first authors

*Joo-Hyun Song: joo-hyun_song@brown.edu

S1 Priming effect comparison

Contents

Fig A in S1 Text. Results comparing full priming and partial priming effect.

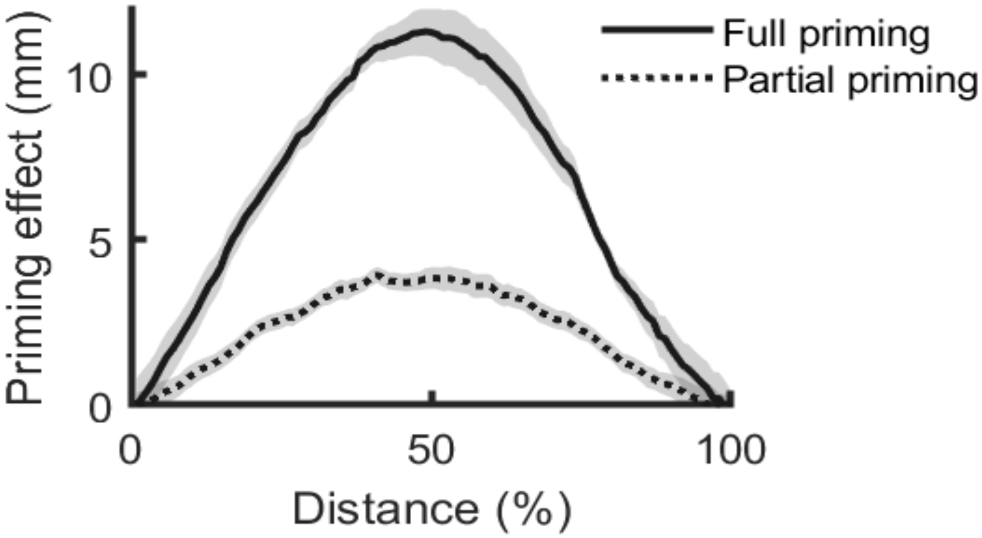

Fig A. Results comparing full priming and partial priming effect. The full priming effect (solid line) was calculated by subtracting the attraction scores in full repeat ($T_R D_R$) condition from attraction scores in full swap ($T_S D_S$) conditions. The partial priming effect (dotted line) was calculated by subtracting the attraction scores in partial repeat conditions from those in partial swap conditions. The shaded region represents the within-subject standard error band. The plot depicts

a stronger priming effect in the case when both target and distractors repeat or swap (full priming), compared with a weaker priming effect when only either target or distractor repeat or swap (partial priming). Specifically, we analyzed whether partial repeat or partial swap (i.e., repeating or swapping only one feature) would demonstrate similar cost-benefits in reaching behavior compared to the full repeat or full swap conditions. Thus, we combined two partial repeats (T_RD_N and T_ND_R) and two partial swaps (T_SD_N and T_ND_S) conditions, respectively, to get the combined representation of partial repeat and partial swap. Although participants initiated (partial repeat: 316.79 + 11.14 vs partial swap: 316.63 + 11.17, $t(20) = 0.194$, $p = .84$, $d = 0.042$) and completed (partial repeat: 810.42 + 20.55 vs partial swap: 812.84 + 20.09, $t(20) = 1.77$, $p = .091$, $d = 0.387$) movements similarly regardless of repeat or swap , we observed that participants moved relatively faster in partial repeat (493.63 + 14.28) compared with partial swap (496.21 + 13.86) conditions ($t(20) = 2.16$, $p = .043$, $d = 0.47$). Participants showed a trend in completing the reach faster in. Participants moved ($t(20) = 3.43$, $p = .003$, $d = 0.74$) as well as completed ($t(20) = 3.52$, $p = .002$, $d = 0.768$) the reach relatively faster in full repeat (489.20 ± 14.04) condition compared to partial repeat (493.63 ± 14.28) condition. Compared to the full conditions, attraction scores in the partial conditions indicate much less deviation toward distractors or a target. Consequently, the priming effect led by two partial conditions is smaller compared with the full conditions. Overall, this is consistent with prior studies with perceptual selection using reaction time measure, demonstrating the existence of partial priming and its relatively smaller strength compared with the full priming [1-4].

Dataset (separate file). See folder “Behavioral Experiment” in https://osf.io/phc6e/files/osfstorage

S1 Text References

1. Eimer M, Kiss M, Cheung T. Priming of pop-out modulates attentional target selection in visual search: Behavioural and electrophysiological evidence. Vision research. 2010 Jun 25;50(14):1353-61.

2. Lamy D, Antebi C, Aviani N, Carmel T. Priming of pop-out provides reliable measures of target activation and distractor inhibition in selective attention. Vision research. 2008 Jan 1;48(1):30-41.

3. Lamy D, Zivony A. Target activation and distractor inhibition underlie priming of pop-out: A response to Dent (this issue). Vision Research. 2018 Aug 1;149:131–8.

4. Dent K. Priming of Pop-out does not provide reliable measures of target activation and distractor inhibition in selective attention: Evidence from a large-scale online study. Vision Research. 2018 Aug 1;149:124-30.
