# Supplementary material for: Continuous action with a neurobiologically inspired computational approach reveals the dynamics of selection history
Source: PLoS Comput Biol. 2023 Jul 17;19(7):e1011283. doi: 10.1371/journal.pcbi.1011283 (PMC10374010; doi:10.1371/journal.pcbi.1011283)
Supplement: S2 Text — Fig A. Factor loadings of PC1. Fig B. The cumulative variance explained by the three PCs across reach distance. Fig C. An illustration of a typical reversal effect between TSDN (medium green) and TNDS (light green) in model 3b (facilitation precedes inhibition) with non-optimal parameters. (PDF) [file pcbi.1011283.s002.pdf]

Supporting Information S2 Text for:

Continuous action with a neurobiologically inspired computational approach reveals the dynamics of selection history

Mukesh Makwana[1,¶], Fan Zhang[2,¶], Dietmar Heinke[2], and Joo-Hyun Song[1]*

[1] Brown University, Providence, Rhode Island, USA

[2] University of Birmingham, Birmingham, United Kingdom

¶ contributed equally and are co-first authors

*Joo-Hyun Song: joo-hyun_song@brown.edu

S2 Principal component regression (PCR) analysis

Contents

Fig A in S2 Text. Factor loadings of PC1.

Fig B in S2 Text. The cumulative variance explained by the three PCs across reach distance.

Fig C in S2 Text. An illustration of a typical reversal effect between T_SD_N (medium green) and T_ND_S (light green) in model 3b (facilitation precedes inhibition) with non-optimal parameters.

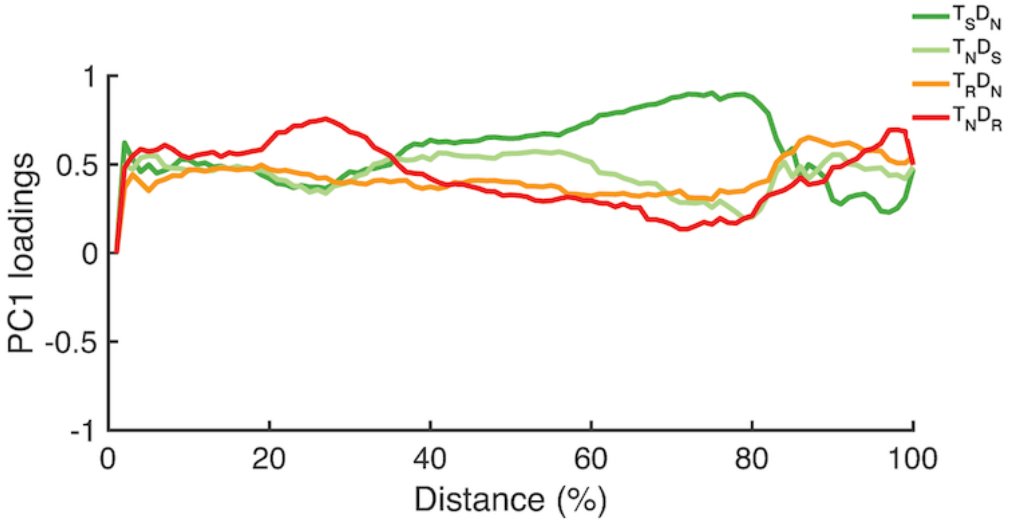

Fig A. Factor loadings of PC1. All four conditions positively loaded on the first principal component (PC1) across the whole reach, which suggests that the first PC represents the common kinematics in the current task regardless of prior history.

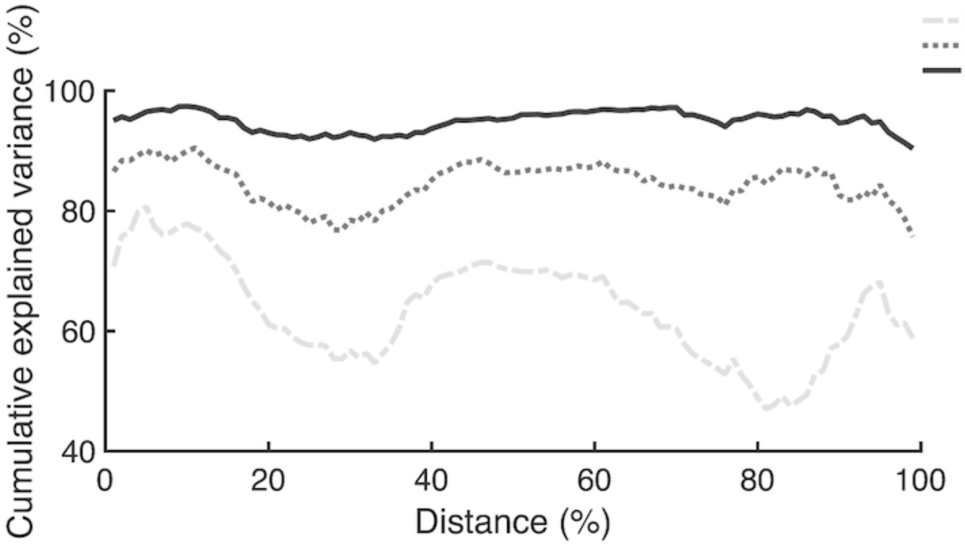

Fig B. The cumulative variance explained by the three PCs across reach distance. The explained variance is calculated based on the eigenvalues of the first three PCs. Altogether they explain more than 95% of the data variance across the reach. The fourth PC, excluded to address the multi-collinearity, has an eigenvalue of 0.05 ± 0.002 averaged across the reach distance.

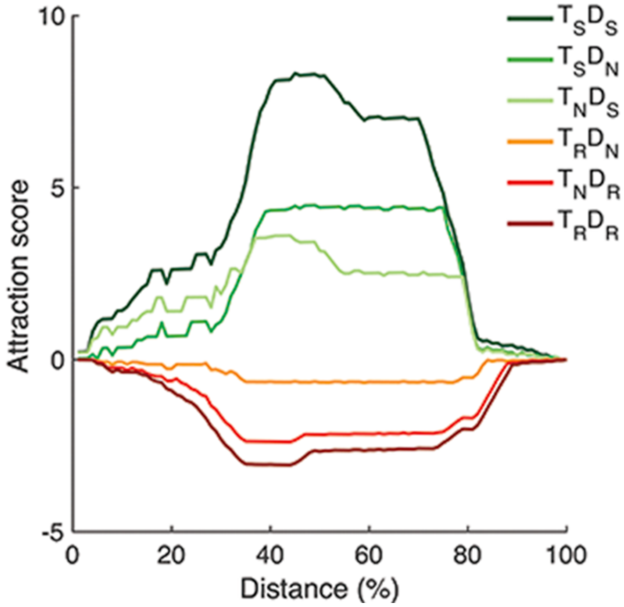

Fig C. An illustration of a typical reversal effect between $T_S D_N$ (medium green) and $T_N D_S$ (light green) in model 3b (facilitation precedes inhibition) with non-optimal parameters.
