# Supplementary material for: Continuous action with a neurobiologically inspired computational approach reveals the dynamics of selection history
Source: PLoS Comput Biol. 2023 Jul 17;19(7):e1011283. doi: 10.1371/journal.pcbi.1011283 (PMC10374010; doi:10.1371/journal.pcbi.1011283)
Supplement: S3 Text — Table A. Parameter setting for color saliencies. Table B. Best fitted model parameters for the Target Selection process. Table C. Parameter ranges of the final grid search in the Target Selection process for the five models. Table D. DNFs parameters in the Movement Production process. S3.A. Target Selection process and mathematical description. S3.B. Selection History module. S3.C. Movement Production process and mathematical description. S3.D. Searching for best parameters. S3.E. Goodness-of-fit. Eq.S1 –S19. (PDF) [file pcbi.1011283.s003.pdf]

Supporting Information S3 Text for:

Continuous action with a neurobiologically inspired computational approach reveals the dynamics of selection history

Mukesh Makwana[1,¶], Fan Zhang[2,¶], Dietmar Heinke[2], and Joo-Hyun Song[1*]

[1] Brown University, Providence, Rhode Island, USA

[2] University of Birmingham, Birmingham, United Kingdom

[¶] contributed equally and are co-first authors

*Joo-Hyun Song: joo-hyun_song@brown.edu

S3 Computational Modeling

Contents

S3.A in S3 Text. Target Selection process and mathematical description.

S3.B in S3 Text. Selection History module.

S3.C in S3 Text. Movement Production process and mathematical description.

S3.D in S3 Text. Searching for best parameters.

S3.E in S3 Text. Goodness-of-fit.

Table A in S3 Text. Parameter setting for color saliencies.

Table B in S3 Text. Best fitted model parameters for the Target Selection process.

Table C in S3 Text. Parameter ranges of the final grid search in the Target Selection process for the five models.

Table D in S3 Text. DNFs parameters in the Movement Production process.

SH-CoR is implemented in MATLAB (version R2019b) [1] using the COSIVINA Toolbox [2]. Note that in the following sections, non-underlined variables are scalars, underlined variables are vectors, and capital letters are matrices.

A. Target Selection process and mathematical description. A detailed architecture of the modules in the Target Selection process is shown in Fig 2. The first stage in Target Selection determines the saliency of the colors in the display (Color Processing). Based on the output of the Color Saliency Layer (Eq. S1), the Odd Color Selection module selects the most salient color and identifies the distractor color and the absent colors via color competition (Eq. S2). These temporal activations are combined with the output of the Feature Maps in a multiplicative way (Eq. S3). This way, the winning color (odd-color) eventually dominates the input to a competition of locations, generating the Target Location Representation (Eq. S4).

Besides color saliency, the Target Selection is also influenced by the Selection History module. This process stores the target and distractor features from the previous trial (Eq. S5-S6) in separated layers. These layers, in turn, influence the selection of the current target through facilitation and/or inhibition depending on which of the five history models are implemented in a particular instantiation of SH-CoR (see schematics in the gray dialog box in Fig 2 for illustration).

Specifically, in the Color Processing module, the Feature Maps ($C_1, C_2\ C_3, C_4$) are decompositions of the input (color display). The Color Saliency Layer combines the saliencies of each color ($c_1^{sal}, c_2^{sal}, c_3^{sal}, c_4^{sal}$) and the stored color features in the History Selection module ($\underline{m}^{SH}$):

$$\underline{I}^{OCS} = (c_1^{sal}, c_2^{sal}, c_3^{sal}, c_4^{sal}) + \underline{m}^{SH} \qquad \text{(Eq. S1)}$$

where $\underline{I}^{OCS}$ represents the input fed into the Color Competition Layer. Note that the saliency of absent colors has the value zero while the target saliency and the distractor saliency are free parameters. Which color saliency, c_1^{sal}, c_2^{sal}, c_3^{sal} or c_4^{sal}, represents absent, target ($\underline{SA_T}$), and distractor items ($\underline{SA_D}$) depends on the condition SH-CoR is set to simulate (see Table A).

Table A. Parameter setting for color saliencies.

	c_1^{sal}	c_2^{sal}	c_3^{sal}	c_4^{sal}
trial (n-1)	SA_T	SA_D	0	0
T_RD_R	SA_T	SA_D	0	0
T_RD_N	SA_T	0	SA_D	0
T_ND_R	0	SA_D	SA_T	0
T_ND_S	0	SA_T	SA_D	0
T_SD_N	SA_D	0	SA_T	0
T_SD_S	SA_D	SA_T	0	0

The Odd Color Selection (OCS) module performs a competition between the neurons using the saliencies as inputs (Color Saliency Layer). The competition is based on Grossberg's recurrent network (RNN) [3]:

$$\tau \cdot \dot{x}_i = a \cdot x_i^{OCS} + b \cdot f(x_i^{OCS}) + c \cdot I_i^{OCS} + d \cdot \sum_l f(x_l^{OCS}) + h + q, \quad f(x_i) = \frac{1}{1+e^{-\beta(x_i - x_0)}} \qquad \text{(Eq. S2)}$$

Where τ is a time constant that defines how fast the neuron adapts toward changing inputs. x_i represents the activation of the *i-th* neuron of the four neurons, and q is a normally distributed noise. The parameter a is a leakage parameter. b and d parameterize local facilitation and a global inhibition (with $d < 0$), respectively. The parameters allow the RNN to generate a range of behaviors (see Table B for the parameter values). Here we configured the RNN to exhibit a winner-take-all (WTA) behavior. A WTA-network activates the unit (i.e., output close to one) with the highest while suppressing all other units (i.e., output close to zero). In SH-CoR, this network detects the most salient color (Odd Color Selection module).

Table B. Best fitted model parameters for the Target Selection process.

		Model 1	Model 2	Model 3a	Model 3b	Model 3c
Color Saliency Layer	Target SA_T	0.55	0.55	0.6	0.55	0.55
	Distractor SA_D	0.35	0.35	0.35	0.30	0.30
Selection History	t_{switch}	-	-	-	7	13
	$\underline{m}_{fac}^{SH}$	0.09	0	0.07	0.07	0.08
	$\underline{m}_{inh}^{SH}$	0	-0.13	-0.13	-0.13	-0.12
Color Competition Layer	a	-0.49	-0.47	-0.47	-0.55	-0.45
	b	1.80	1.85	1.7	1.75	1.80
	c	0.45	0.35	0.35	0.40	0.45
	d	-2.50	-2.45	-2.55	-2.55	-2.60

The output of the Feature Maps (C_1, C_2, C_3, C_4) is multiplied with the output of the Odd Color Selection module (Π (pi) operator in Fig.2) and form the input of the Target Location Map (TLM):

$$I^{TLM} = (f(x_1^{OCS}(t)), f(x_2^{OCS}(t)), f(x_3^{OCS}(t)), f(x_4^{OCS}(t)) \cdot (C_1, C_2, C_3, C_4)' \quad \text{(Eq. S3)}$$

Eventually, the target location is computed in the Target Location Map (x_{ij}^{TLM}) as the Target Location Representation $f(T^{TLM})$:

$$\tau \cdot \dot{x}_{ij}^{TLM} = -x_{ij}^{TLM} + I_{ij}^{TLM} + h_{loc}; \quad f(x) = \frac{1}{1+e^{-\beta(x-x_0)}} \quad \text{(Eq. S4)}$$

B. Selection History module

The output activation of the Odd Color Selection module is thresholded (see Table C) and stored in the Target Feature Layer (TFL):

$$x_i^{TFL}(t) = \begin{cases} f(x_i^{OCS}(t)) & f(x_i^{OCS}(t)) < \theta \\ 1 & f(x_i^{OCS}(t)) \geq \theta \end{cases} \quad \text{(Eq. S5)}$$

Table C. Parameter ranges of the final grid search in the Target Selection process for the five models.

		Range					
Color Saliency Layer	Target SA_T	0.65	0.6	0.55			
	Distractor SA_D	0.25	0.3	0.35			
	Absent	0					
Selection History	t_{switch}	7	9	11	13	15	17
	θ	0.9					
Color Competition Layer	a	-0.55	-0.53	-0.51	-0.49	-0.47	-0.45
	b	1.70	1.75	1.80	1.85		
	c	0.35	0.40	0.45			
	d	-2.60	-2.55	-2.50	-2.45		
	β	9					
	h	2					
	x_0	0.3					
	τ	50					
	q	0.005					

In addition, models 2 and 3a, 3b, and 3c require the detection of the distractor. A biologically plausible implementation for this detection is beyond the scope of the research question in this paper. Therefore, we set the value of the Distractor Feature Layer (DFL) depending on the condition SH-CoR aims to simulate. The output of the Selection History module is the weighted summation of the two layers:

$$\underline{m}^{SH} = \underline{m}_{fac}^{SH} \cdot \underline{x}^{TFL} + \underline{m}_{inh}^{SH} \cdot \underline{x}^{DFL} \quad \text{(Eq. S6)}$$

The exact implementation of this equation depends on the selection history model SH-CoR is set to simulate. For model 1, $\underline{m}_{inh}^{SH}$ is set to zero and for model 2 $\underline{m}_{fac}^{SH}$ is set to zero. Model 3a, both parameters are set to non-zero values. To implement model 3b and model 3c, an additional parameter, the switch time t_{switch} (see S2 Table), was added. This parameter determines at which time the inhibition is switched on and facilitation is switched off (model 3b) and vice versa (model 3c).

C. Movement Production process and mathematical description.

Movement Production process receives target location information from the Target Selection process and produces the reaching movement to the target. The model implements Dynamic Neural Fields (DNFs) Theory [2,4]. This theory proposes that neural activation in two-dimensional layers acts as a representation of movement parameters (e.g., target location, movement velocity, etc.).

The Displacement Representation (*D*) is created through a 2D convolution of Hand Location Representation (H^{LOC}) with the Target Location Representation $f(T^{TLM})$:

$$D(\underline{x}, t) = \int H^{LOC}\left(\underline{y} - \underline{x},\ t\right) \cdot f(T^{TLM}(\underline{x}))d\underline{x} \qquad \text{(Eq. S7)}$$

whereby H^{LOC} is a 2D Gaussian function to represent the hand location:

$$H^{LOC}(\underline{x}, t) = A \cdot e^{-\frac{(\underline{x} - \underline{x}(t))^2}{2\sigma^2}} \qquad \text{(Eq. S8)}$$

where x and y represent the hand position as a function of time, in each simulated time step, we update the hand position (reach movement) based on the hand velocity in a close control loop (see Fig. 2). The Displacement Representation (*D)* forms the input into the Velocity Representation together with a 2D Gaussian function of the same size as H^{LOC} but does not move as a function of time:

$$I^{Vel1}(\underline{x}, t) = D(\underline{x}, t) + A \cdot e^{-\frac{\underline{x}^2}{2\sigma^2}} \quad \text{(Eq. S9)}$$

The addition of the Gaussian function ensures a build-up of activation in the Velocity Representation at the beginning of a simulation run. This blob-like activation (see Fig. 3 for an illustration) forms the basis for the moving blob. The Velocity Representation uses a two-layer DNF (layer $Vel1$ and layer $Vel2$) to generate the moving blob [4-6]. Layer $Vel1$ consists of lateral interactions with separate local excitatory and inhibitory plus global inhibitory connections:

$$\tau \cdot \dot{x}_i^{Vel1} = -x_i^{Vel1} + I^{Vel1} + h + exc_{loc}(x_i^{Vel1}) + inh_{loc}(x_i^{Vel1}) + inh_{glob}(x_i^{Vel1}) + q \quad \text{(Eq. S10)}$$

To stabilize the moving blob, Layer $Vel1$ feeds into Layer $Vel2$ via local excitatory, inhibitory plus global inhibitory connections, analogous to the recurrent connection in Layer $Vel2$:

$$\tau \cdot \dot{x}_i^{Vel2} = -x_i^{Vel2} + h + exc_{loc}(f(x_i^{Vel1})) + inh_{loc}(f(x_i^{Vel1})) + inh_{glob}(f(x_i^{Vel1}) + q) \quad \text{(Eq. S11)}$$

The excitatory and inhibitory components are defined with the following Equations:

$$exc_{loc}(\underline{x}) = \int w_{exc}(\underline{x} - \underline{x}') \cdot f(\underline{x}')d\underline{x}' \qquad \text{(Eq. S12)}$$

$$inh_{loc}(\underline{x}) = \int w_{inh}(\underline{x} - \underline{x}') \cdot f(\underline{x}')d\underline{x}' \qquad \text{(Eq. S13)}$$

$$inh_{glob}(x) = g_{inh} \cdot \int f(x')dx' \qquad \text{(Eq. S14)}$$

in which the kernel w with strength c and width parameter σ is defined with:

$$w(\underline{x}) = \frac{c}{\sigma\sqrt{2\pi}} \cdot exp(-\frac{|\underline{x}|^2}{2\sigma^2}) \qquad \text{(Eq. S15)}$$

See Table D for the parameters in the above-mentioned Equations for the Movement Production process.

D. Searching for best parameters. To find the best fitting parameter settings for each of the five models, we had to employ some heuristics since simulation runs of SH-CoR are very time-consuming. First, we used a grid search [7] across a range of parameter values (Table C) with a deterministic version of SH-CoR (see Table B for the result) using the goodness-of-fit function defined below. Then we re-run SH-CoR with noise added 21 times. The repeats reflect the number of participants in the behavioral experiment. This additional step established the robustness of the best fits. The fixed parameter and the range of free parameters varied in the grid search can be found in S3 Table. Note that since these alternative selection history mechanisms differently influence the Target Selection process, grid search did not only search parameters for the Selection History module (Eq. S6) but also for the Color Competition layer (Eq. S2) and Color Saliency Layer (Eq. S1), which include both the target saliency (SA_T) and distractor saliency (SA_D). The parameters for the motor production process are kept constant during the grid search (Table D).

Table D. DNFs parameters in the Movement Production process.

	τ	β	h	A	σ	g_{inh}	c_{exc}	σ_{exc}	c_{inh}	σ_{inh}
T^{LOC}	40	4	-30							
H^{LOC}				7	6					
D				5						
Vel1	20	4	-5			-0.01	5	5	0	10
Vel2	5	4	-0.2			-0.005	5	5	0.8	10

E. Goodness-of-fit. The goodness-of-fit for this search is based on two terms. The first error term begins with calculating the mean squared differences between model attraction scores and the human attraction scores averaged across all distances for each condition and then summing these averages to obtain a total error characterizing the mismatch between human data and model behavior:

$$e_1 = \sum_k^6 \sum_{j=11}^n (x_{kj} - y_{kj})(x_{kj} - y_{kj})' \qquad \text{(Eq. S16)}$$

x_{tj}: simulated attraction scores at each distance point (j: 11% - 90%, $n = 90$),
y_{tj}: human attraction scores at each distance point (j: 11% - 90%),
and k refers to the six experimental conditions (k=1: T_RD_R; k=2: T_ND_R; k= 3: T_RD_N; k= 4: T_ND_S; k=5: T_SD_N; k=6: T_SD_S).

The second error term (e_2) is motivated by the observation that the size of the attraction scores (Fig.1C) for all distances is ordered in the following way: $T_RD_R < T_ND_R < T_RD_N < T_ND_S < T_SD_N < T_SD_S$. The second error term punishes model results that violate this order:

$$e_2 = \sum_k^5 \sum_{j=11}^n f(x_{kj} - x_{(k+1)j}) \qquad \text{(Eq. S17)}$$

$$f(x_{kj} - x_{(k+1)j}) = \begin{cases} 0 & (x_{kj} - x_{(k+1)j}) < 0 \\ 1 & (x_{kj} - x_{(k+1)j}) \geq 0 \end{cases} \qquad \text{(Eq. S18)}$$

To sum up, we have the overall evaluation of the model performance as the error term*:*

$$e = log_{10}(e_1 + s \cdot e_2) \qquad \text{(Eq. S19)}$$

where s is a scaling factor for e_2 (*s=50*).

S3 Software (separate file). See folder "*SH-CoR*" in https://osf.io/phc6e/files/osfstorage

S3 Text References

1. MATLAB. version 9.7 (R2019b). Natick, Massachusetts: The MathWorks Inc.; 2019 Sep 11.

2. Erlhagen W, Schöner G. Dynamic field theory of movement preparation. Psychological Review. 2002;109(3):545–72.

3. Grossberg S. Recurrent neural networks. Scholarpedia. 2013 Feb 22;8(2):1888.

4. Amari SI. Dynamics of pattern formation in lateral-inhibition type neural fields. Biological cybernetics. 1977 Jun;27(2):77-87.

5. Strauss S, Woodgate PJ, Sami SA, Heinke D. Choice reaching with a LEGO arm robot (CoRLEGO): the motor system guides visual attention to movement-relevant information. Neural Networks. 2015;72:3–12.

6. Faubel C, Schöner G. Learning to recognize objects on the fly: a neurally based dynamic field approach. Neural networks. 2008 May 1;21(4):562-76.

7. Kochenderfer MJ, Wheeler TA. Algorithms for optimization. Mit Press; 2019 Mar 12.
